# Progress Toward Digital Transformation in an Evolving Post-Acute Landscape

**DOI:** 10.1093/geroni/igac021

**Published:** 2022-04-06

**Authors:** Dori A Cross, Julia Adler-Milstein

**Affiliations:** Division of Health Policy and Management, University of Minnesota School of Public Health, Minneapolis, Minnesota, USA; Department of Medicine, University of California San Francisco, San Francisco, California, USA; Center for Clinical Informatics and Improvement Research, University of California San Francisco, San Francisco, California, USA

**Keywords:** Health information technology, Interoperability, Transitions of care

## Abstract

Digitization has been a central pillar of structural investments to promote organizational capacity for transformation, and yet skilled nursing facilities (SNFs) and other post-acute providers have been excluded and/or delayed in benefitting from the past decade of substantial public and private-sector investment in information technology (IT). These settings have limited internal capacity and resources to invest in digital capabilities on their own, propagating a limited infrastructure that may only further sideline SNFs and their role in an ever-evolving health care landscape that needs to be focused on age-friendly, high-value care. Meaningful progress will require continuous refinement of supportive policy, financial investment, and scalable organizational best practices specific to the SNF context. In this essay, we lay out an action agenda to move from age-agnostic to age-friendly digital transformation. Key to the value proposition of these efforts is a focus on interoperability—the seamless exchange of electronic health information across settings that is critical for care coordination and for providers to have the information they need to make safe and appropriate care decisions. Interoperability is not synonymous with digital transformation, but a foundational building block for its potential. We characterize the current state of digitization in SNFs in the context of key health IT policy advancements over the past decade, identifying ongoing and emergent policy work where the digitization needs of SNFs and other post-acute settings can be better addressed. We also discuss accompanying implementation considerations and strategies for optimally translating policy efforts into impactful practice change across an ever-evolving post-acute landscape. Acting on these insights at the policy and practice level provides cautious optimism that nursing home care—and care for older adults across the care continuum—may benefit more equitably from the promise of future digitization.

**Translational Significance:** Policy and health system efforts to advance digitization have fallen short of meeting the needs of older adults across the care continuum. Improving the design, implementation, and use of health information technology (IT) in skilled nursing facilities (SNFs)—with an emphasis on the interoperable exchange of information to support transitions in and out of this care environment—represents a critical step toward age-friendly digital investment. We identify key areas of enabling policy action and strategies to mitigate organizational challenges that hinder the translation of enabling policy into meaningful IT-enabled environments for SNFs and across the post-acute ecosystem.

Among the many fronts on which the U.S. health care system is pursuing transformational solutions to achieve more affordable, accessible, and high-value care, the centerpiece of the last decade is digitization (1). Significant public and private-sector investment has been devoted to achieving widespread digitization of health records alongside adoption and use of digital tools that support care delivery. With sustained attention to design, implementation, and continuous improvement, there are many domains where this investment is expected to pay off. In settings with robust electronic health record (EHR) systems, patient safety can be enhanced with computerized entry and checking of medication orders (2–4). Population health advancements can be fueled by clinical data analytics that identify gaps in care and underlying disparities (5–7). Patients have better access to their medical information to help them better understand their health status and the actions they can take to improve it (8,9). More recently, sophisticated algorithms driven by artificial intelligence are predicting clinical trajectories and tailored interventions (9). These examples represent exciting advancements, yet the net payoff from investment in health information technology (IT) has been modest and required substantially more effort and investment than anticipated.

Digitization, it turns out, is the easy part. Digital transformation is a much more complex and incremental undertaking—one in which the technologies are not the limiting step and instead stem from the broader organizational changes necessary to implement and use technology in impactful ways (10). When people, policies, and processes are not well aligned with the technologies, improvements take longer and fall short of the expected value. These issues are particularly apparent in lower-resourced settings that struggle to afford advanced IT tools as well as invest in the complementary people, policy, and process changes (6,11–13). The vision of an integrated, fully IT-enabled health care delivery system still has substantial momentum behind it. However, meaningful progress will require continuous refinement of supportive policy, financial investment, and scalable best practices for implementation and sustained change.

As we head into the second decade of work that marks a transition from pursuing digitization to achieving digital transformation, the priorities and levers of change are harder to pinpoint. We can no longer rely on prior measures of progress. Federal investment programs that have supported adoption and use of EHRs to date offered a ladder of progression from structural capabilities to demonstrated use of enhanced capabilities. These efforts were limited to hospitals and eligible office-based settings, and offered significant flexibility to accommodate different provider settings’ capacity and needs. To keep advancing our goals, we need to expand these efforts to identify and address the most salient design, implementation, and use issues that continue to impede progress toward optimal IT-enabled care—especially for the patient populations most expected to benefit.

Meeting the care needs of older adults is a critical area to focus these ongoing and future efforts. This diverse population exhibits a range of care needs as they age. Compared to younger age groups, they are more likely to have complex care needs and interact with many types of health care delivery settings that need to be coordinated—spanning from inpatient settings to home-based care. They are also more likely to have family caregivers involved in their health and health care management, and may require accommodation for different levels of digital literacy and digital interest. As a result, digitally supported care for older adults requires not only that they benefit equitably from generalized investments already made in IT capabilities, but also that specific capabilities are designed and tailored to meet their unique needs (14). There is an urgent need to characterize and address current gaps where older adults could benefit more fully from digitally supported care.

In this paper, we focus on the interoperable sharing of information across sites of care as critical for supporting the care of older adults. Care transitions, especially upon hospital discharge, often lack the robust information sharing practices necessary to ensure that follow-up providers have the information they need to make timely and appropriate care decisions (ie, reconciliation of medications, follow-up testing needs, etc.) (15,16). Investing in electronic health information exchange (HIE) to facilitate data exchange can help. Use of HIE supports reductions in inefficiency and improvements in patient safety (17). While early focus was on the electronic sending and receiving of patient information, interoperability is the key policy goal expected to more reliably add value to patient care delivery (18). Interoperability establishes additional benchmarks around the ability to search for and integrate outside information into the local data system of an end user (see Figure 1). Without the ability to access information within existing workflows, providers are unlikely to be aware of or make use of external data resources (19–21).

**Figure 1.**
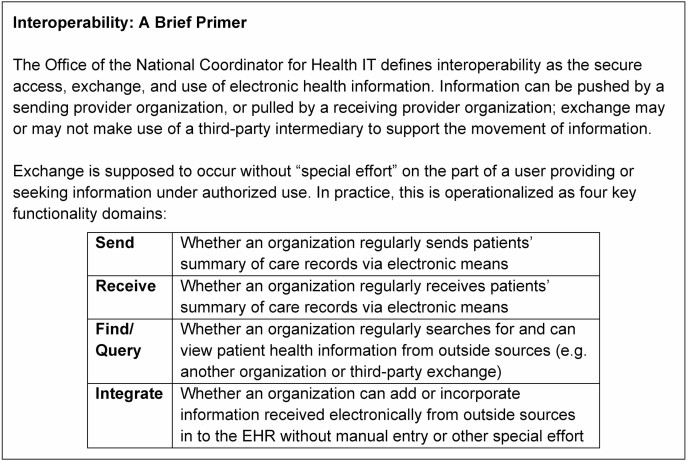
Defining interoperability. EHR = electronic health record; IT = information technology.

Policy efforts continue to prioritize the advancement of interoperability through structural improvements (updated unified data standards and common frameworks for exchange) as well as HIE-focused performance measures under value-based payment programs (18,22–24). And yet, interoperability has been challenging to advance in policy and in practice due to a range of technical, legal/regulatory, economic, and governance challenges (11,25,26). Interoperability—and the IT infrastructure that enables these capabilities—continues to be lacking in settings and contexts most relevant to the care needs of older adults (27–29). This remains true despite the fact that older adults are especially vulnerable to the gaps and errors that result from poor information sharing during transitional care (30–32). Thus, moving forward, interoperability advancements need to more explicitly focus on the care needs and continuum of care for older adults when establishing broader goals and expectations for digital transformation efforts.

This paper focuses on a key setting of care for older adults—skilled nursing facilities (SNFs). Focusing on SNFs provides a useful example of the specific design and implementation considerations for age-friendly interoperability advancements. Specific information needs to support patients’ clinical and social care needs, as well as the resource-challenged context in which digital advancements must be made, suggest that challenges and opportunities in this setting are likely to translate to older adults’ needs and experiences across the continuum of care. First, we summarize key contextual factors driving SNF needs for digitization and interoperability. Next, we briefly describe current data characterizing the current state of adoption of computerized systems and electronic health information sharing in SNFs. We then review key health IT policy advancements over the past decade, and identify areas of ongoing policy work where the digitization needs of SNFs and other post-acute settings can be better addressed. We also discuss accompanying implementation considerations and strategies for optimally translating policy efforts into impactful practice change. Identifying and addressing implementation challenges in the SNF environment, particularly with respect to resource needs that foster an environment conducive to organizational change, has never been more important given the sustained impact of coronavirus disease 2019 (COVID-19) in these settings (33,34). Finally, we address key ways in which the post-acute care landscape is changing. Continued trends toward use of home-based services in coordination with or in lieu of institutional skilled nursing care necessitate a broader conversation that includes digitization for home health agencies (HHAs) and other caregiver-reliant home- and community-based services. Acting on these insights at the policy and practice level promotes the prioritization of nursing home care—and care for older adults across the care continuum—as we continue to invest in optimizing digitization.

## Current SNF Care Environment

### Transitional Care Needs and Challenges

Nursing homes play a critical role in the care continuum, including short-stay SNF care as a critical and high-volume “throughway” for hospitalized patients on their way to recovery and return to community. Transitions into and out of SNF care represent significant disruption and leave patients and their families vulnerable to gaps, miscommunications, and errors in care (19,31,32). Over 3 million patients annually are hospitalized and then discharged to an SNF (35). Upon SNF admission, information sharing is known to be incomplete, delayed, and difficult to use (36). This creates challenging workflows and an environment where SNF staff do not always have the right information and resources on hand to provide safe, appropriate, and patient-centered care. For example, facilities might not have had enough advance notice to have the correct wound care supplies on hand, or might upset the patient if unaware of key aspects of family history (eg, recent death of a spouse). These care disruptions lead to patient discomfort, compromised care quality in the nursing facility, and increase the risk of avoidable emergency department (ED) visits or rehospitalizations (31,37,38).

SNF discharge practices to send patients home are similarly strained and poorly coordinated. Between 2011 and 2017, the percentage of SNF patients discharged home rather than to long-term nursing home care rose steadily from 33% to 40% and is expected to keep rising (39). These transitions are difficult, as patients are experiencing 2 transitions in relatively short succession (ie, hospital to SNF and SNF to home) (40). Successful transitions back to the community require coordinating with the providers who will take over care for the individual. Medical providers may need to be updated, or the individual may need a new primary care provider. There may be new need for home health care, durable medical equipment, and outpatient rehabilitation. Timely sharing of relevant information about when the individual is being discharged from SNF care and subsequent care management needs is critical, but sorely lacking (41,42). Primary care providers, as well as other payer or health system entities responsible for care coordination, often do not even know when a patient is in an SNF, let alone have the ability to access information that supports transitional care processes such as knowledge of pending orders and authorizations, updated medication lists, and assessment of a patient’s living environment and social support (43,44). Resulting care gaps and lack of patient and family support often destabilize patients such that they end up in the ED or rehospitalized soon after SNF discharge (40).

### Pressure on SNFs From Value-Based Payment

Post-acute services are a significant driver of the total cost of care, and reflect highly variable levels of utilization and care quality (39). Payment and delivery reforms are therefore increasingly targeting post-acute care by shifting to financial risk arrangements that span these services. These include both accountable care and bundled payment contracting, as well as readmission-based penalties incorporated directly into standard hospital and SNF reimbursements (45,46). Hospitals discharging patients have increasingly strong incentives to optimize transitional care. And yet, responding to these changing incentives is challenging. Hospitals that participate in Medicare Accountable Care Organizations are incentivized to discharge patients sooner, enabled by the Medicare 3-day waiver which allows these hospitals to bypass the required 3-day hospital stay typically required before a patient is eligible for transfer to an SNF. This means that hospitals need transitional care practices that support that transition of increasingly vulnerable and unstable patients. Hospitals are indeed investing more, though unevenly, in strengthened coordination with SNF referral partners (47). Many hospitals lack sufficient exposure to value-based payment to motivate these care delivery changes; those that do often focus their investment of time and resources for transitional care quality improvement only with select facilities (19,47,48). This means that only a percentage of SNFs could be expected to benefit from these investments, and the incentives under value-based payment only indirectly influence digital advancement. Organizations care more about improved patient outcomes, but currently receive no explicit expectations or guidance on how to invest in IT-enabled transitional care that is most effective in this context.

SNF discharge practices for patients returning to the community are also under increased scrutiny in payment redesign. Facilities are facing new incentives to discharge patients back to the community sooner, and to ensure that these are safe and well-coordinated transitions. These expectations are only expected to strengthen, despite the fact that SNFs have fewer staff, technology, and resources to invest in transitional care relative to hospital discharge programs (38,40). This underresourcing of SNFs poses broader challenges to fostering an environment of learning and quality improvement—IT-related and beyond. Recently synthesized evidence demonstrates that knowledge translation—bringing evidence into practice—is particularly challenging in SNFs and other long-term or post-acute settings and impedes individual and organizational-level change (48). Motivated leadership, engaged staff, and organizational slack (ie, the extra energy and resources that facilitate goal-setting/innovation beyond basic organizational obligations) are critical components of organizational capacity for digital advancement. However, a highly regulated environment as well as chronic underpayment and understaffing lead to unstable leadership, high staff turnover, and chronic gaps in the types of institutional knowledge and motivation needed to improve the status quo. The ongoing effects of the COVID-19 pandemic have only amplified these long-standing issues (49).

## Current State of Digitization in SNFs

Against this backdrop it is therefore not surprising that SNFs, and the broader set of long-term and post-acute care providers, reflect a substantial degree of variability with respect to their adoption and use of EHRs, interoperability solutions, and other digital tools. Recent representative surveys estimate that EHRs have been adopted in at least two-thirds of SNFs (28). Interestingly, SNF EHR adoption has not been found to be associated with organizational size (eg, number of beds) or rurality, but has been found to be negatively associated with for-profit status and ownership by a multifacility chain (28,50). The extent to which these ownership structures inhibit facility-level organizational innovation has been proposed as 1 key mechanism explaining this disparity in adoption and use (50).

EHR-enabled SNFs most often utilize computerized functions related to clinician notes and medication management (ie, recording and reconciling medications), and lag substantially in interoperability capabilities. Over 40% of facilities surveyed in 2018 had zero interoperability capabilities (ie, send, receive, query, and/or integrate), and an additional 25% had only one of these functions in use (50). Because of the hospital-to-SNF transition use cases driving interoperability in the post-acute context, SNFs are more likely to be able to receive (41.3%) or query for (31.7%) information than to send information to outside partners (22%). Integration capabilities—where information from outside sources is automatically pulled into the SNF’s EHR—remain extremely low (12.4%). Perhaps as a result, in a recent national survey, SNFs often report that, following hospital transitions, key information is missing or delayed. Even information considered fundamental (ie, contact info for discharging provider, notice of pending test results) was reported to be often missing among at least 20% of respondents, and over half of respondents noted that discharge summaries were often not available at the time of patient transfer (34).

## Federal Health IT Policy and Opportunities for Tailoring to SNFs

Current levels of SNF digitization, and opportunities for progress, are best understood in the broader context of health system digitization efforts. The 2009 Health IT Economic and Clinical Health (HITECH) Act EHR incentive programs tied Medicare and Medicaid dollars to increasingly advanced demonstrations of EHR “Meaningful Use.” However, these incentives were only made available to eligible hospitals and eligible professionals, definitions that largely excluded SNFs and other types of short- and long-term post-acute care providers. Thus, as EHR adoption and maturity of EHR systems increased following the HITECH Act programs, this increase was only experienced by eligible providers, with clear evidence that ineligible providers remained on a slower path toward digitization and digital maturity (13,51).

In many ways, it is therefore remarkable that, without any federal incentives, the majority of SNFs have been able to adopt EHRs and use them to support core functions like clinical documentation and medication management. However, the exclusion from Meaningful Use put these providers on a different trajectory that involved implementation of different setting-specific EHRs that were not subject to the same data standards and certification criteria as those in acute care settings. Using the same data standards across organizations is fundamental to the ability to transport and integrate data from one setting to another. Further, many of the advanced EHR functions—such as clinical decision support—that have been adopted in acute care settings are still rare in SNFs. It is not clear what is needed to close these gaps. It is possible that the shift to value-based payment models will create new motivation for investment in EHRs among the one-third of SNFs that have not yet digitized, which are disproportionately those that are for-profit and part of a multifacility chain. However, if adoption rates stagnate, policymakers will need to identify the barriers as well as the facilitators to achieve broad adoption of at least basic EHR systems.

Given the key role of SNFs in the care continuum, it is particularly critical that we focus not only on internal IT capabilities but also building capacity to engage in robust information sharing—both receiving information when patients are admitted and sending information when patients are discharged. It is concerning that, even among SNFs that have adopted EHRs, the ability to easily receive and send information electronically is limited. HITECH fell short in advancing information sharing to support post-acute care not only by making SNFs ineligible for the Meaningful Use program, but also not offering any guidance for participating hospitals to focus on exchange with SNFs. As a result of these policy design decisions, eligible hospitals and eligible professionals had little incentive to customize their approach to electronic information sharing with SNFs, that is, ensuring that documentation met SNF informational needs and workflows for receiving and integrating received data (27,52). This, in turn, allowed varied approaches to facilitating information sharing at the time of SNF transitions—ranging from traditional phone/fax/patient carried to varied electronic approaches that could accommodate different levels of SNF digitization (eg, giving the SNF the ability to view the hospital record via a portal, connecting to a regional electronic health information sharing network) (50,53). For SNFs, this means that they face varied choices about how to share information without a single solution that creates connectivity to all needed partners.

Since passage of HITECH, there have been several complementary policy efforts, notably under the 21st Century Cures Act, to increase interoperability across the care continuum (22). The hope is that these efforts will address existing gaps in SNF digitization—particularly in their ability to electronically exchange information and integrate external data into local systems. For example, through 2021, states can request enhanced matched Medicaid funds for efforts to get long-term and post-acute provider settings connected to regional information exchange networks, but SNFs participation in these HIE structures has historically been so low (<20%) that stronger efforts are needed (28). There are many options for such efforts and they are not mutually exclusive. We suggest focus on the following 3 options:

*As part of ongoing meaningful use criteria, require that eligible hospitals demonstrate connectivity to key long-term and post-acute care (LTPAC) partners.* Hospitals are key partners with SNFs in the continuum of care and their approach to information sharing during discharge to SNFs has a significant influence on how well SNFs can support the transition (19,29,31,32). Yet hospitals have no requirements to send information to SNFs electronically, even when they are capable of doing so. Adapting Meaningful Use criteria to direct hospitals to electronically share transitional care information in advance of discharge would directly motivate hospitals to invest in the processes and technologies to improve information sharing during this critical care transition. Such criteria may benefit from further tailoring to SNFs. For example, early access to the types of information that SNFs need to prepare for patient arrival, timely completion and transmission of discharge summaries, and/or use of SNF-specific templates that prioritize the ordering of information relevant to SNF admission processes could help promote information sharing processes that prioritize SNF needs (34).
*Expand data and document standards to specifically support common care transitions for older adults, including hospital to SNF.* Ideally, eligible hospitals would not need to individually customize their EHRs to produce documentation that includes the key information SNFs need and format that information in a clear and usable way. Here, new data and document standards might help promote more consistent completeness and usability of information that is exchanged in the post-acute context. Under the 21st Century Cures Act, hospital EHRs are now required to capture data using a common language and make it available for exchange via a common architecture (22). The common architecture is Application Programming Interfaces (APIs) that are widely used in other industries and enable core data operations: create, read, update, and delete. The common language that these APIs will use to enable interoperability of health information is FHIR—a draft standard describing data formats and elements (eg, medication, immunization). Further, hospital EHRs are required to make specific types of data available via FHIR-based APIs (the U.S. Core Data for Interoperability, or USCDI). USCDI will expand the breadth of data elements included over time, creating a natural opportunity to ensure that the specific types of information relevant to older adults in general (eg, caregiver status) and specifically to those making hospital–SNF transitions (eg, functional status, cognitive status) are included. Data specifications to support these transfers are still in development as part of the ONC 360X collaborative workgroup (54). Even though vendors in these settings are not subject to the same certification criteria through which USCDI standards are enforced, this work is facilitated by parallel data standard expectations under the Impact Act of 2014. These efforts tend to progress slowly given disproportionate attention on digital transformation in hospitals and office-based physicians. However, the COVID-19 pandemic has foregrounded plans for how to build capacity to rapidly adapt USCDI to include emergent types of critical data, and this includes a necessary focus on post-acute care (55).
*Build and invest in health IT use cases to drive SNF quality.* Because SNFs were not eligible for HITECH programs, they not only missed out on the financial incentives and ability to adopt certified EHR systems but also missed out on the organizational transformation and learning about people and process change to advance digital transformation. Advancing quality within SNFs relies on helping facilities understand how to leverage data and IT tools in pursuit of broader goals, such as fewer errors, enhanced patient engagement, improved public health reporting, and seamless care transitions (6,10,53). Indeed, prior literature suggests that technical capabilities alone are not sufficient to drive routine use and better outcomes in the SNF setting (19,56). Thus, for SNFs to truly close the gap in both digitization and digital transformation, they need targeted financial support and technical assistance—essentially, their own EHR incentive program that improves upon the lessons learned during HITECH. Any type of policy or program should explicitly include interorganizational learning and collaboration both across SNFs and community-based efforts between SNFs and the hospitals, community-based physicians, HHAs, etc. with whom they share responsibility for the total care continuum.

## Digital Transformation in an Evolving Post-Acute Care Landscape

For SNFs, successful digital transformation would be marked by immediate, real-time access to hospital medical records that supports patients care with timely and complete information. However, we cannot focus only on hospital–SNF interoperability. A plan for age-friendly digital investment needs to be comprehensive with respect to who is involved (eg, clinicians, patients, family) and where they are reached—that is, across the full continuum of clinical and social care services, and at home. More specifically, exciting areas of development include:

*Active caregiver engagement with the digital health ecosystem.* Family caregivers are critical in supporting the health and health care needs of older adults, and effective caregiver involvement has been linked to improved health outcomes (57). State-based efforts under the CARE Act require hospitals to identify and document caregivers in the EHR, and contact/provide education to these individuals at the time of hospital discharge (58). This is a critical first step toward facilitating change around consistent caregiver inclusion and ultimately expanding these expectations to other sites of care, such as SNFs. Yet, health systems struggle to help patients achieve these benefits by meaningfully integrating family caregivers as part of the care team. Caregiver proxy access to patient record portals continues to grow, but evidence of appropriate and supported use has not (14,59,60). Increased experience with restricted visitation between older adult patients and their caregivers as a result of the COVID-19 pandemic may attract greater interest in proxy use. However, to really advance digitally enabled caregiver engagement, we need an increased policy push on developers to improve functionality of patient and caregiver-facing portals and applications (ie, with respect to user-centered design and integration). We also need health systems to see the value of age-friendly health services, and invest more purposefully in the culture change and process redesign necessary to see any meaningful change in the patient experience as a result of these tools being available.
*A focus on at-home health care.* The ongoing shift in referrals toward home health utilization, rather than SNFs and other institutional post-acute care, means that these organizations are increasingly critical to digitize (61,62). While HHAs have higher rates of EHR adoption and self-reported interoperability capabilities compared to SNFs, functionality is still highly variable (28). These organizations are also more likely to be using multiple methods of exchange, which creates challenging workflows and can lead to inconsistent use of interoperability features even when they are readily available. Numerous opportunities exist to advance HHA interoperability and other digital capabilities alongside that of SNFs—in particular around remote patient monitoring and connectivity to community-based social services, using many of the same levers around payment and technology policies.

## Conclusion

The future of IT and information sharing capabilities in long-term and post-acute care settings is promising but uncertain. The COVID-19 pandemic has only underscored the critical need for digitization and digital transformation of SNFs and other post-acute providers. Doing so requires more focused and direct policy attention to generate the resources, incentives, and organizational capacity needed to implement industry-level change. Value-based payment and delivery reforms may help as health systems direct more attention and resources to their partners across the continuum of care, but are not sufficient on their own. To truly direct progress from age-agnostic to age-friendly digital transformation, we must actively advocate for these identified action areas—for example, improved data standards and implementation support for tailored use cases, digitally inclusive development of advanced IT functionalities—that center the needs of older adults in an ever-changing post-acute care environment.
